# The epidemiology and societal costs of myasthenia gravis in Norway: A non‐interventional study using national registry data

**DOI:** 10.1111/ene.16233

**Published:** 2024-02-07

**Authors:** Ingrid Engebretsen, Nils Erik Gilhus, Ivar Sønbø Kristiansen, Erik Magnus Sæther, Ingrid Lindberg‐Schager, Fredrik Arneberg, Christoffer Bugge

**Affiliations:** ^1^ Oslo Economics Oslo Norway; ^2^ Department of Clinical Medicine University of Bergen Bergen Norway; ^3^ Department of Neurology Haukeland University Hospital Bergen Norway; ^4^ Department of Health Management and Health Economics, Institute of Health and Society University of Oslo Oslo Norway; ^5^ Department of Public Health, Research Unit for General Practice University of Southern Denmark Odense M Denmark; ^6^ UCB Pharma AB Stockholm Sweden; ^7^ UCB Pharma AS Oslo Norway

**Keywords:** cost of illness, epidemiology, healthcare costs, myasthenia gravis, productivity loss

## Abstract

**Background and purpose:**

With the emergence of new treatment options for myasthenia gravis (MG), there is a need for information regarding epidemiology, healthcare utilization, and societal costs to support economic evaluation and identify eligible patients. We aimed to enhance the understanding of these factors using nationwide systematic registry data in Norway.

**Methods:**

We received comprehensive national registry data from five Norwegian health‐ and work‐related registries. The annual incidence and prevalence were estimated for the period 2013–2021 using nationwide hospital and prescription data. The direct, indirect (productivity losses) and intangible costs (value of lost life‐years [LLY] and health‐related quality of life [HRQoL]) related to MG were estimated over a period of 1 year.

**Results:**

In 2021, the incidence of MG ranged from 15 to 16 cases per year per million population depending on the registry used, while the prevalence varied between 208.9 and 210.3 per million population. The total annual societal costs of MG amounted to EUR 24,743 per patient, of which EUR 3592 (14.5%) were direct costs, EUR 8666 (35.0%) were productivity loss, and EUR 12,485 (50.5%) were lost value from LLY and reduced HRQoL.

**Conclusion:**

The incidence and prevalence of MG are higher than previously estimated, and the total societal costs of MG are substantial. Our findings demonstrate that productivity losses, and the value of LLY and HRQoL constitute a considerable proportion of the total societal costs.

## INTRODUCTION

Myasthenia gravis (MG) is a rare neuromuscular disease which manifests as varying degrees of skeletal muscle weakness [1]. It is an autoimmune disorder in which antibodies block or destroy acetylcholine receptors in the postsynaptic membrane at the neuromuscular junction. Patients commonly experience fatigue, ocular symptoms (double vision and/or ptosis), problems with swallowing and speech, and weakness in the neck, shoulders and arms. Some individuals may also encounter MG crises characterized by respiratory muscle weakness, requiring respiratory support and intensive care [1].

In milder cases of MG, acetylcholinesterase inhibitors may be sufficient to manage the disease. However, most patients require additional treatment with corticosteroids and immunosuppressants [2, 3]. Thymectomy is performed for early‐onset, generalized MG with acetylcholine receptor antibodies. Intravenous immunoglobulin and plasma exchange represent widely used short‐term treatments for MG exacerbations or when rapid improvement is needed [2, 3]. Present standard treatments lead to a good outcome in approximately 80% of MG patients, whereas 20% are regarded as difficult to treat. Short‐ and long‐term side effects and long latency before optimal response represent challenges in current MG treatment. With the increasing financial burden on healthcare systems worldwide, cost‐effectiveness analyses are becoming necessary to ensure optimal allocation of available resources. Information on epidemiology, resource utilization, and the broader societal costs is needed to feed the mathematical simulation models used to evaluate the cost‐effectiveness of new treatments. Information is also essential for policymakers to understand how individual patients are affected by the disease and to assess equity of care and resource utilization.

Estimates of MG incidence and prevalence vary across countries [2, 4, 5]. Prevalence rates are rising in most countries, underscoring the need for updated information to estimate population costs. Additionally, information about healthcare utilization and patient costs for MG is lacking. A recent review highlighted the limited availability of data, primarily restricted to small geographical areas and specific cost categories [6]. To our knowledge, no previous study has comprehensively described the broader societal costs of MG and encompassed all relevant categories within a nationwide geographic setting. Norway possesses excellent health registries capturing data on all 5.5 million of its residents. The universal public healthcare system and publicly financed hospitals provide a solid foundation for assessing the total patient population without selection bias. Health registers allow estimation of total resource utilization and disease‐related costs.

In this study, we utilize nationwide registry data to assess the epidemiology, healthcare resource utilization, and societal costs of MG in Norway. Our data enable us to estimate the aggregated societal costs of MG, encompassing direct healthcare costs, productivity losses, and the value of lost quality‐adjusted life‐years.

## METHODS

### Study design

We conducted a registry‐based cohort study of patients with MG based on nationwide, compulsory registry data.

### Data sources

We obtained individual patient‐level data from 2008 to 2021 from the Norwegian Patient Registry (NPR), the Norwegian Prescription Database (NorPD) and the Norwegian Primary Care Database (KUHR), as well as aggregated data from the Norwegian Welfare and Labor Administration (NAV; 2012–2020) and the Norwegian Cause of Death Registry (NCDR; 1996–2019). The NPR captures data on all treatment episodes in publicly funded hospitals (in‐ and outpatient care, including in‐hospital drug use), while the NorPD contains data on all pharmacy‐dispensed prescription drugs, including all symptomatic and immunosuppressive MG drugs available in Norway during the study period. The KUHR (Control and Payment of Health Reimbursement) holds data on treatments given by general practitioners (GPs), at local emergency rooms, and by private practice specialists.

**FIGURE 1:**
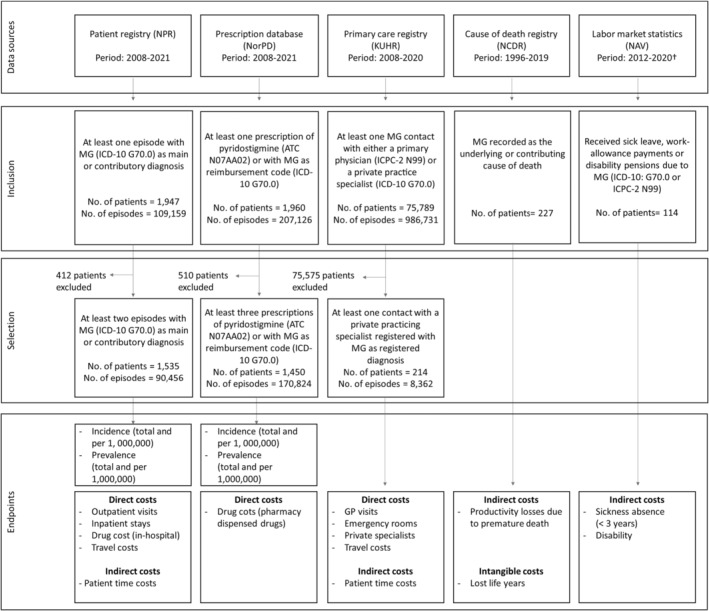
Overview of data sources, inclusion criteria, patient selection, and endpoints. †Data on disability pensions were only available until 2016. ATC, Anatomical Therapeutic Chemical; GP, general practitioner; ICD‐10, International Classification of Diseases, 10th revision; ICPC‐2, International Classification of Primary Care, 2nd edition; KUHR, Norwegian Primary Care Database; MG, myasthenia gravis; NAV, Norwegian Welfare and Labor Administration; NCDR, Norwegian Cause of Death Registry; NorPD, Norwegian Prescription Database; NPR, Norwegian Patient Registry.

Patients, and their encounters with the healthcare service, were identified in the registries based on diagnostic codes derived from the International Classification of Diseases, 10th revision (ICD‐10 code: G70.0) and the International Classification of Primary Care, 2nd edition (ICPC‐2 code: N99; Figure 1). Validation of diagnostic codes in the NPR has been conducted in previous research in other disease areas, and diagnostic codes for cancer are found to be valid when compared to the Cancer Registry of Norway [7]. Prescription drugs were identified based on the Anatomical Therapeutic Chemical (ATC) codes, including patients with pyridostigmine prescriptions (ATC code N07AA02). Data from the NAV included information on the number of patients with MG diagnosis receiving a disability pension and MG‐related welfare payments. Additionally, data on the number of MG patients and payments for sickness absence (<3 years) due to other neurological diseases (ICPC‐2 code N99) were obtained from the NAV. The NCDR provided data on the number of deaths attributed to MG, stratified by underlying and contributing causes of death [8].

The data were collected from each registry separately and were not linked.

### Identification of MG patients

From the NPR, we defined MG patients as all individuals with at least two hospital encounters with an MG diagnosis (ICD‐10 G70.0). From the NorPD, individuals were classified as MG patients if they had at least three MG‐specific drug prescriptions (pyridostigmine) or three MG‐specific reimbursement codes (ICD‐10 G70.0). These criteria were chosen because a diagnostic code may be recorded by error, or the purpose of an encounter may be assessment of MG. For the KUHR, we selected patients with an encounter with a private practice specialist registered with the code ICD‐10 G70.0. Due to the lack of an MG‐specific code in the ICPC‐2 diagnostic system used by GPs, the registry data from the KUHR could not be used to estimate MG incidence or prevalence.

Several individuals were likely to be present in multiple registries. Unfortunately, due to lack of registry linkage, we were unable to track individual patients across registries.

### Study endpoints

#### Incidence and prevalence

Incidence and prevalence (total and per 1,000,000) were estimated separately from the NPR and NorPD. To identify incident MG patients, we employed a ‘wash‐out period’ of 5 years, defined as the period from 1 January 2008 to 31 December 2012. We considered patients with no MG‐related encounters during this period as newly diagnosed cases. Prevalence was estimated as the number of patients previously diagnosed with MG who were alive by the end of each year. Annual population data used to estimate incidence and prevalence rates were obtained from Statistics Norway [9].

#### Societal costs

Societal costs were assessed and categorized into three groups, as outlined by Drummond et al. [10]: direct costs, indirect costs, and intangible costs.

##### Direct costs

Costs that could be completely attributed to MG were estimated by including only costs of MG‐related treatment episodes, identified by using the diagnostic codes recorded for each patient episode in each registry. Direct costs encompassed primary care (GP and emergency room visits), private practice specialists, hospital treatment, prescription drugs, and patient travel costs. All MG‐related drug prescriptions were included in the prescription drug cost analysis. MG‐related drug prescriptions for our sample of MG patients were identified by ATC codes (Table S6). We used Diagnosis Related Group (DRG) cost weights, reimbursement fees, patient co‐payments, and drug prices (excluding VAT), following a methodology similar to that used by Bugge et al. [11]. Patient travel costs associated with treatment were derived from the literature [12] and adjusted for inflation. Treatment episodes unrelated to MG were excluded from the calculation of direct costs.

##### Indirect costs

Productivity losses were estimated using the human capital approach [13] as well as data on welfare payments from the NAV. In Norway, people can be certified sick for up to 52 weeks receiving their full salary, and then refused payments for a certain period of time. These two schemes are limited to 3 years in total. After 3 years, patients may receive disability pension. For sickness absences (<3 years), we assumed that 2.5% of patients recorded with the ICPC‐2 code for ‘other neurological diseases’ (N99) received welfare payments due to MG, based on aggregated data on disability pensions from the NAV. To reflect the actual societal cost of work absenteeism, welfare payments were adjusted according to the wages of the beneficiaries and adjusted for societal and overhead costs according to Norwegian guidelines [14]. Patients' time costs were estimated for the prevalent population using data from the NPR and KUHR and unit costs obtained from the literature [12]. We assumed 2 h of lost leisure time for each primary care visit, 3 h for outpatient hospital visits, 8 h per day for inpatient care, and 20 min for telephone consultations. For tax‐financed services (direct costs and welfare payments), we assumed that the added marginal costs of using public funds would constitute 20% of all MG‐related expenditures covered by the public, according to guidelines from the Norwegian Ministry of Finance [14]. Productivity losses related to informal care (care from family, friends) or reduced working ability when at work (presenteeism) were not included due to a lack of reliable data.

##### Intangible costs

Intangible costs encompass benefits foregone that have no direct impact on the consumption or production of resources. Intangible costs encompass concepts such as pain and suffering. We estimated the intangible costs as the value of lost life‐years (LLYs) and of lost health‐related quality of life (HRQoL) attributable to MG. The value of LLYs is related to the loss of quality‐adjusted life years (QALYs) due to premature death, whereas lost HRQoL represents the reduction in life quality for those living with MG. Age‐specific MG‐related deaths in 1996–2019 from the NCDR and expected remaining QALYs in the general population from the Norwegian Medicines Agency [15] were used to estimate LLYs. We accounted for the HRQoL of the remaining life‐years by comparing age of death among MG patients (NCDR data) with the estimated age‐specific expected remaining QALYs in the general population. For instance, a patient aged between 65 and 69 years was expected to have 14.9 remaining QALYs according to national guidelines [15]. If this patient died from MG, the LLY will be 14.9 QALYs. Information on HRQoL for MG patients was obtained from the literature [16] and compared to the general population [15]. Intangible costs (LLY and HRQoL) were monetized according to national guidelines based on EUR 140,000 per lost QALY [17]. All costs were estimated for the year 2020 using a prevalence‐based costing approach [13]. The estimates were converted from 2020 Norwegian Kroner (NOK) to Euros (EUR) using the average exchange rate for 2022 (1 EUR = 10.7 NOK).

### Statistical analyses

We undertook descriptive analyses only. Analyses were conducted using R version 4.1.2 (2021), STATA software version 15 (College Station, TX, USA), and Microsoft Excel (2016).

### Ethics

Approval to use data from the NPR and NorPD were obtained from the National Research Ethics Committees (REK; 2021/102). Approval to use data from the KUHR, NAV and NCDR was provided by the individual registry holder.

## RESULTS

From 2008 to 2021, a total of 1947 patients had at least one hospital treatment episode recorded with a diagnostic code for MG (Figure 1). Among them, 1535 were classified as confirmed MG patients according to our predefined criteria. A total of 1960 patients received at least one prescription of pyridostigmine or at least one prescription with MG reimbursement code in the period 2008–2021. Of these, 1450 individuals were classified as MG patients. Furthermore, 214 patients were in contact with a private specialist for their MG between 2008 and 2020. Due to a lack of linked data we did not know whether these were the same patients as those identified in the hospital and prescription data.

### Incidence and prevalence

The incidence of MG in 2021 was estimated to be 86 (16.0 per million) when using hospital data (NPR), and 81 (15.0 per million) when using prescription data (NorPD; Table 1). The estimated incidence varied from 13.7 per million inhabitants in 2014 to 18.8 per million in 2020 (hospital data). When using prescription data, the incidence ranged from 11.5 per million in 2014 to 18.2 per million in 2020. As for MG prevalence, in 2021 it was estimated to be 1126 (208.9 per million) based on hospital data and 1134 (210.3 per million) based on prescription data.

**TABLE 1:** Incidence and prevalence of myasthenia gravis by year in Norway, 2013–2020 (total and per 1,000,000).

	Number of new cases				Number of patients alive by 31 December each year			
	Based on hospital data		Based on prescription data		Based on hospital data		Based on prescription data	
Year	Total	Per million	Total	Per million	Total	Per million	Total	Per million
2013	83	16.4	71	14.1	719	142.3	687	136.0
2014	70	13.7	59	11.5	760	148.8	717	140.3
2015	91	17.6	74	14.3	832	161.1	772	149.4
2016	93	17.8	86	16.5	900	172.6	834	160.0
2017	87	16.5	86	16.4	964	183.3	900	171.2
2018	94	17.8	96	18.1	1029	194.3	975	184.1
2019	100	18.8	97	18.2	1093	205.1	1036	194.4
2020^a^	90	16.8	95	17.7	1141	212.6	1096	204.2
2021^a^	86	16.0	81	15.0	1126	208.9	1134	210.3

Abbreviations: NorPD, Norwegian Prescription Database; NPR, Norwegian Patient Registry.

^a^ Incidence estimates from 2020 and 2021 may be affected by restricted access to healthcare services during the pandemic.

### Direct costs

The total direct costs associated with MG in 2020 amounted to EUR 4.10 million, equivalent to EUR 3592 per patient, representing 14.5% of the total societal costs of MG (Table 2). Hospital care accounted for the majority of direct costs, totaling EUR 3.57 million (87.1%), with inpatient treatment contributing with EUR 3.16 million (88.5%). In 2020, 572 patients received inpatient treatment for MG (50.1% of all MG patients). The costs of prescription drugs were estimated at EUR 380,000 (EUR 335 per patient), while primary care costs amounted to EUR 50,000 (EUR 44 per patient). The most common drugs used for MG treatment in Norway are shown in Table S3. Patient travel costs were estimated to be EUR 80,000 (EUR 75 per patient). Hospital‐related costs per patient in 2020 varied widely among patients (Figure S1, supplementary material). The mean MG‐related costs of inpatient care were EUR 15,386, with a standard deviation of EUR 19,266.

**TABLE 2:** Total societal costs of myasthenia gravis in Norway in 2020.

	Total costs, million 2020 EUR	Cost per patient^a^, 2020 EUR
**Direct costs**	**4.10**	**3592**
Primary care visits	0.05	44
Hospital treatment	3.57	3131
Outpatient care visits	0.42	367
Inpatient care visits	3.16	2764
Private practice specialist visits	0.01	8
Prescription drugs	0.38	335
Patient travel costs	0.08	75
**Indirect costs (productivity loss)**	**9.89**	**8666**
Sickness absence (<3 years)	1.09	958
Disability	5.99	5250
Patient time costs	1.46	1258
Marginal costs of public funds (distortions in the labour market)	1.36	1199
**Intangible costs (value of LLYs and HRQoL)**	**14.24**	**12,485**
Nonfatal health losses	4.63	4056
Value of lost life‐years	9.62	8428

Abbreviations: HRQoL, health‐related quality of life; LLY, lost life‐years. The total cost for each cost category is highlighted in bold.

^a^ Cost per patient was estimated using point prevalence 31.12.2020 from the Norwegian Patient Registry (*N* = 1141).

### Indirect costs

The productivity loss due to MG in 2020 was estimated at EUR 9.89 million (EUR 8666 per patient), constituting 35.0% of the total societal costs related to MG (Table 2). Overall, 12.7% of the patients received disability pension due to their MG (Table S1, supplementary material), resulting in an average productivity loss of EUR 41,338 per disabled patient per year. The average cost of sickness absence from paid work (<3 years) was EUR 17,418 per year for those receiving sickness payments. Patient time costs were estimated at EUR 1258 per patient. The marginal cost of public funds, which accounts for distortions in the labour market from income taxation, was estimated to be EUR 1.36 million (EUR 1199 per patient).

### Intangible costs

The intangible costs accounted for 50.5% of the total societal costs related to MG (Table 2). Society values the fact that people are alive and in good health, and we express this value in monetary terms. Health conditions that lead to death or reduced quality of life impose an intangible cost to society. In total, 227 patients died with MG recorded as an underlying or contributing cause of death between 1996 and 2019, representing 9.5 deaths per year (Table S2, supplementary material). By comparing age‐specific MG‐related deaths to the expected remaining QALYs for the general population by age group, we estimate that MG‐related deaths caused 73.5 lost QALYs per year in 2020. Of the estimated QALY loss, 88.9% was related to excess mortality among patients aged 60 years and over. The annual average lost HRQoL for MG patients collected from the literature was 0.22 QALYs [16], compared to an annual loss of 0.19 QALYs for the general population. The MG‐related HRQoL was thus estimated as 0.03 QALYs, resulting in a total loss of 34.4 QALYs per year for the prevalent MG population. Using national guidelines to monetize the health losses, the values of LLY and lost HRQoL were estimated as EUR 8428 (0.06 QALYs) and EUR 4056 (0.03 QALYs) per patient per year, respectively.

## DISCUSSION

The incidence of MG in Norway in 2021 was 15–16 cases per million, while the prevalence was 208.9–210.3 per million. The robustness of these estimates was proven by using data from two separate and independent nationwide registries, providing consistent results. The total annual societal cost associated with MG amounted to EUR 24,743 per patient, of which EUR 14.5% stemmed from direct costs, 35.0% came from productivity loss, whereas 50.5% came from the value of LLYs and HRQoL.

In industrialized countries, reported incidence rates of MG have ranged from 4 to 30 cases per year per million population, while prevalence has been reported between 150 and 200 cases per million [18]. For Norway, previous research has indicated an annual incidence of approximately 10 cases per million and a prevalence of 145 per million [4, 5]. Our study indicates that MG in Norway is more frequent than reported previously. Our results correspond well to international estimates based on recent data [18, 19], but our estimated prevalence is somewhat lower that what is reported in recent studies from Germany (393 per million) and Spain (260 per million) [19, 20]. We do not know whether MG has become more common, if some cases had remained undiagnosed previously, or if previous studies missed some diagnosed cases. Regardless of explanation, we believe that our data provide more accurate estimates than previous studies because the estimates are based on updated nationwide registry data spanning across multiple years. We found a somewhat lower incidence of MG in the NorPD than the NPR. Several factors may have contributed to this difference. Firstly, not all MG patients are necessarily prescribed patient‐administered MG drugs. Additionally, physicians may sometimes prescribe MG‐related drugs without using the appropriate reimbursement code. Further, primary care physicians use ICPC‐2 N99 reimbursement codes for prescriptions. Prescriptions recorded with an ICPC‐2 N99 diagnostic code were not used for estimates of incidence, because it was not possible to distinguish MG patients from other disease groups captured by ICPC‐2 N99.

In terms of economic costs related to MG, our study adds important information to a sparse body of evidence. As highlighted in a recent systematic review [6], former studies ignore several important cost categories. Our findings clearly show that indirect and intangible costs, often omitted from previous studies of MG, represent large proportions of the total societal MG costs. Compared to the societal costs for other diseases, the productivity loss related to MG accounts for a relatively high proportion of the total societal costs. While we estimate that the productivity losses account for 35.5% of the MG costs, the corresponding proportion for all diseases taken together is estimated by the Norwegian Directorate of Health to account for 9.5% of the total societal costs [21]. The direct healthcare costs of MG represent 0.011% of total healthcare expenditure in Norway [22]. Such estimates for other countries have not been published. We estimate the cost per MG patient per year at EU 24,743, while similar estimates are EUR 84,894 for cancer [11] and EUR 7583 for migraine [23]. MG patients in Norway have a relatively high share of inpatient stays compared to the number of primary care visits and outpatient stays at hospitals. With an MG exacerbation or with a comorbidity (infection or other), the threshold is probably low for hospitalization. Further, the estimates of primary care visits are likely underestimates as their classification system (ICPC‐2) does not contain a specific diagnostic code for MG.

A key strength of this study lies in the nationwide data spanning multiple years. Having a universal public healthcare system, where a total national population receives healthcare in publicly financed hospitals, implies that the registries capture all individuals residing in the country, eliminating problems with selection bias and lack of representativeness. This enables us to describe the epidemiology of the disease and estimate costs that can be attributed to MG with high precision as very few MG patients receive care in the private sector. Current knowledge on how MG influences the ability to work is limited. Another strength of this study is that the National Insurance Scheme is nationwide and includes diagnostic information which allows us to identify welfare payments received by beneficiaries due to MG specifically.

Our study has some limitations. Working with unlinked registry data represents a challenge in identifying MG patients and may result in double‐counting of patients. This is especially relevant for the data on sickness absence where we used the unspecific ICPC‐2 code N99 to identify MG patients. The NAV maintains separate data records for sickness absence and disability pensions. The data related to disability pensions offer a higher level of detail, including ICD‐10 diagnosis information, enabling the calculation of payments specifically attributed to MG among the larger group of ‘Diseases of the nervous system’. When it comes to sickness absence, physicians only register the ICPC‐2 code ‘Other neurological diseases’. However, the costs of sickness absence only account for 11% of the indirect costs, while disability (for which we have precise data based on ICD‐10 codes) accounts for more than 60% of the costs. Hospital encounters may be registered with an MG diagnostic code by mistake, or MG may be misdiagnosed, and physicians may prescribe pyridostigmine to patients without MG. However, diagnostic codes in the NPR are found to be valid when checked against other disease‐specific quality registries [24]. Whereas a first MG diagnostic code can be entered while waiting for confirmation of the diagnosis, the final diagnostic sensitivity and specificity for MG is reported to be high [5]. To avoid overestimation of the number of patients and total population costs, we applied strict criteria to identify MG patients that should favour specificity over sensitivity. We used a definition of at least two MG diagnostic codes or three prescriptions of pyridostigmine, which will limit the risk of overestimation. Several criteria for defining MG patients were investigated as a part of the study (Supplementary Tables S4 and S5). The criteria used to identify MG patients may result in patients with milder MG being excluded from the study. However, most patients will be referred to a hospital, preferably the outpatient clinic, for assessment and treatment if MG is suspected. The NPR also captures patients treated at outpatient clinics and our expectation is that the long‐term follow‐up will enable us to capture the vast majority of MG patients in Norway, even those with mild disease. MG‐related mortality was based on death certificates with suboptimal validity because autopsies are rarely performed in Norway. The completed death certificate should show a logical sequence from the underlying to the immediate cause of death to be valid [8], but in a regional Norwegian study, 32% of death certificates had an illogical structure [25]. Due to the reporting of age in grouped intervals rather than precise figures in some registries and a limited number of total deaths, age‐group‐specific MG death rates were not deemed robust enough to be presented in the study. This also implies uncertainty in estimation of intangible costs.

Some important MG‐related societal costs were not considered in our study due to the unavailability of data. This includes informal care costs and lost productivity due to reduced working ability. In the case of other disease areas, such as cancer, informal care costs have been estimated to account for 10.1% of the total productivity losses [11]. Costs related to reduced working ability have been estimated to account for 39.5% of the productivity losses related to migraines [23].

Multiple new treatments for MG are becoming available [26]. These treatments are associated with high costs, and their costs and benefits need to be evaluated prior to public or private funding. The current treatment costs of MG described in this study can encourage policymakers and health authorities to include cost‐effectiveness analyses to support reimbursement decisions. Changes in treatment costs as well as changes in outcome for MG patients need to be assessed, as described in this study. There is an ongoing debate as to whether all costs beyond the healthcare sector, such as productivity losses, should be included in cost‐effectiveness analyses evaluating new treatments [27]. As highlighted in our study, these costs represent a considerable proportion of the total societal costs of MG. Of the patients in this study, 12.7% received disability pension due to their MG. Society will benefit from limiting the number of patients who fall out of the workforce. Interventions to reduce the burden on public welfare schemes should have a high priority.

In this study, we present resource use and costs per patient. These estimates represent the mean for all MG patients, including both patients with mild disease and those with the most severe forms of MG, and independent of age or working status. Previous studies indicate that costs of MG vary widely by patient characteristics [16]. In the case of direct medical costs, treatment with intravenous immunoglobulin or with plasma exchange are important cost drivers [16]. Future research should be aimed at understanding costs and resource use of different subpopulations of MG, including those with severe MG, to support the economic evaluations and treatment decisions regarding for whom and when to introduce new treatment options.

In conclusion, MG represents a significant burden on both patients and society, not least in terms of LLY, lost HRQoL, and lost productivity. Information on the broader societal cost of MG could assist policymakers in budgeting and planning, serve as a useful basis for priority setting, and provide knowledge for comparative analyses across countries and healthcare systems.

## FUNDING INFORMATION

This study was funded by UCB Pharma.

## CONFLICT OF INTEREST STATEMENT

Ingrid Engebretsen, Ivar Sønbø Kristiansen, Erik Magnus Sæther, and Christoffer Bugge are affiliated with Oslo Economics and have all completed consultancy assignments for several pharmaceutical companies in recent years. Ingrid Lindberg‐Schager and Fredrik Arneberg work at UCB Pharma. Nils Erik Gilhus has received financial support from UCB, Argenx, Janssen, Merck, Roche, Alexion and Immunovant.

## Supporting information


**Appendix S1.** Supporting Information.

## Data Availability

The data that support this study are not publicly available due to ethical restrictions.

## References

[1] Gilhus NE . Myasthenia gravis. NEJM. 2016;375(26):2570‐2581. 28029925 10.1056/NEJMra1602678

[2] Gilhus NE , Tzartos S , Evoli A , Palace J , Burns TM , Verschuuren J . Myasthenia gravis. Nat Rev Dis Primers. 2019;5(1):30. 31048702 10.1038/s41572-019-0079-y

[3] Narayanaswami P , Sanders DB , Wolfe G , et al. International consensus guidance for Management of Myasthenia Gravis: 2020 update. Neurology. 2021;96(3):114‐122. 33144515 10.1212/WNL.0000000000011124 PMC7884987

[4] Heldal AT , Owe JF , Gilhus NE , Romi F . Seropositive myasthenia gravis: a nationwide epidemiologic study. Neurology. 2009;73(2):150‐151. 19597135 10.1212/WNL.0b013e3181ad53c2

[5] Andersen JB , Engeland A , Owe JF , Gilhus NE . Myasthenia gravis requiring pyridostigmine treatment in a national population cohort. Eur J Neurol. 2010;17(12):1445‐1450. 20491896 10.1111/j.1468-1331.2010.03089.x

[6] Landfeldt E , Pogoryelova O , Sejersen T , Zethraeus N , Breiner A , Lochmüller H . Economic costs of myasthenia gravis: a systematic review. PharmacoEconomics. 2020;38(7):715‐728. 32363541 10.1007/s40273-020-00912-8

[7] Bakken IJ , Gystad SO , Christensen ØO , et al. Comparison of data from the Norwegian patient register and the cancer registry of Norway. Tidsskr nor Laegeforen. 2012;132(11):1336‐1340. 22717858 10.4045/tidsskr.11.1099

[8] Pedersen AG , Ellingsen CL . Data quality in the causes of death registry. J Norwegian Med Assoc. 2015;135:768‐770. 10.4045/tidsskr.14.1065 25947599

[9] Statistics Norway . Table 07459: Population, by sex and one‐year age groups (M) 1986–2023. 2023.

[10] Drummond MF , Sculpher MJ , Claxton K , Stoddart GL , Torrance GW . Methods for Economic Evaluation of Health Care Programmes. 4th ed. Oxford University Press; 2015.

[11] Bugge C , Sæther EM , Brustugun OT , Kristiansen IS . Societal cost of cancer in Norway ‐results of taking a broader cost perspective. Health Policy. 2021;125(8):1100‐1107. 34088521 10.1016/j.healthpol.2021.05.008

[12] Moger TA , Kristiansen IS . Direct and indirect costs of the Norwegian breast cancer screening program. HERO. 2012;2012:3.

[13] Jo C . Cost‐of‐illness studies: concepts, scopes, and methods. Clin Mol Hepatol. 2014;20(4):327‐337. 25548737 10.3350/cmh.2014.20.4.327 PMC4278062

[14] Norwegian Ministry of Finance . Prinsipper og krav ved utarbeidelse av samfunnsøkonomiske analyser. Ministry of Finance; 2014.

[15] The Norwegian Medicines Agency . Guidelines for the submission of documentation for single technology assessment (STA) of pharmaceuticals (Updated 2021). 2018. Accessed June 29, 2023. https://legemiddelverket.no/english/public‐funding‐and‐pricing/documentation‐for‐sta/guidelines‐for‐the‐submission‐of‐documentation‐for‐single‐technology‐assessment‐sta‐of‐pharmaceuticals

[16] Mendoza M , Tran C , Bril V , Katzberg HD , Barnett C . Patient‐acceptable symptom states in myasthenia gravis. Neurology. 2020;95(12):e1617‐e1628. 32759200 10.1212/WNL.0000000000010574

[17] The Norwegian Directorate of Health . Samfunnskostnader ved sykdom og ulykker 2013 – sykdomsbyrde, helsetjenestekostnader og produksjonstap fordelt på sykdomsgrupper. The Norwegian Directorate of Health; 2016.

[18] Dresser L , Wlodarski R , Rezania K , Soliven B . Myasthenia gravis: epidemiology, pathophysiology and clinical manifestations. J Clin Med. 2021;10(11):2235. 10.3390/jcm10112235 PMC8196750 34064035

[19] García Estévez DA , López Díaz LM , Pardo Parrado M , et al. Epidemiology of myasthenia gravis in the province of Ourense (Galicia, Spain). Neurología (English Edition). 2023;38(2):75‐81. 10.1016/j.nrleng.2020.06.013 35249845

[20] Mevius A , Jöres L , Biskup J , et al. Epidemiology and treatment of myasthenia gravis: a retrospective study using a large insurance claims dataset in Germany. Neuromuscul Disord. 2023;33(4):324‐333. 36921445 10.1016/j.nmd.2023.02.002

[21] The Norwegian Directorate of Health . Samfunnskostnader ved sykdom og ulykker 2015. 2019.

[22] Statistics Norway . Health accounts. Accessed. https://www.ssb.no/en/nasjonalregnskap‐og‐konjunkturer/nasjonalregnskap/statistikk/helseregnskap. 2022.

[23] Oslo Economics . Migrene i et samfunnsperspektiv . 2020.

[24] Bakken I , Gystad S , Christensen Ø , et al. Comparison of data from the Norwegian patient register and the cancer registry of Norway. J Norwegian Med Assoc. 2012;132:1336‐1340. 10.4045/tidsskr.11.1099 22717858

[25] Alfsen CG , Lyckander LG , Lindboe AW , Svaar H . Quality control of deaths in hospital. J Norwegian Med Assoc. 2010;130:476. 10.4045/tidsskr.09.0744 20224612

[26] Schneider‐Gold C , Gilhus NE . Advances and challenges in the treatment of myasthenia gravis. Ther Adv Neurol Disord. 2021;14:17562864211065406. 34987614 10.1177/17562864211065406 PMC8721395

[27] Yuasa A , Yonemoto N , LoPresti M , Ikeda S . Use of productivity loss/gain in cost‐effectiveness analyses for drugs: a systematic review. PharmacoEconomics. 2021;39(1):81‐97. 33230613 10.1007/s40273-020-00986-4 PMC7790765

