# Supplementary material for: The epidemiology and societal costs of myasthenia gravis in Norway: A non‐interventional study using national registry data
Source: Eur J Neurol. 2024 Feb 7;31(5):e16233. doi: 10.1111/ene.16233 (PMC11235824; doi:10.1111/ene.16233)
Supplement: Supplementary file 1 — Appendix S1. Supporting Information. [file ENE-31-e16233-s001.docx]

**SUPPLEMENTARY DATA**

*Table S1: Resource utilization for MG patients in Norway, year 2013-2020*

|  | 2013 | 2014 | 2015 | 2016 | 2017 | 2018 | 2019 | 2020 |
| --- | --- | --- | --- | --- | --- | --- | --- | --- |
| **Direct resource utilization** |  |  |  |  |  |  |  |  |
| **Primary care visits** |  |  |  |  |  |  |  |  |
| Total number of episodes | 569 | 524 | 679 | 610 | 648 | 700 | 550 | 557 |
| Average no. of episodes per patient in contact with primary healthcare | 6.9 | 5.7 | 7.2 | 6.0 | 6.2 | 7.1 | 5.4 | 5.8 |
| Average no. of episodes per MG patient | 0.8 | 0.7 | 0.8 | 0.7 | 0.7 | 0.7 | 0.5 | 0.5 |
| **Private specialist visits** |  |  |  |  |  |  |  |  |
| Total number of episodes | 75 | 82 | 62 | 75 | 52 | 62 | 64 | 53 |
| Average no. of episodes per patient in contact with private practicing specialists | 1.7 | 1.8 | 1.7 | 1.7 | 1.5 | 1.6 | 1.5 | 1.9 |
| Average no. of episodes per MG patient | 0.1 | 0.1 | 0.1 | 0.1 | 0.1 | 0.1 | 0.1 | 0.0 |
| **Hospital visits** |  |  |  |  |  |  |  |  |
| Total number of out-patient visits | 859 | 965 | 1,095 | 1,273 | 1,366 | 1,524 | 1,541 | 1,734 |
| Total number of in-patient stays | 574 | 480 | 426 | 558 | 688 | 678 | 597 | 572 |
| Average no. of out-patient visits per patient in contact with outpatient | 1.9 | 2.1 | 2.2 | 2.5 | 2.4 | 2.5 | 2.6 | 2.6 |
| Average no. of out-patient stays per MG patient | 1.2 | 1.3 | 1.3 | 1.4 | 1.4 | 1.5 | 1.4 | 1.5 |
| Average no. of in-patient stays per patient in contact with in-patient | 2.9 | 2.7 | 2.4 | 2.7 | 3.3 | 3.1 | 3.0 | 2.8 |
| Average no. of in-patient visits per MG patient | 0.8 | 0.6 | 0.5 | 0.6 | 0.7 | 0.7 | 0.5 | 0.5 |
| **Prescription drugs** |  |  |  |  |  |  |  |  |
| Total number of pyridostigmine prescriptions | 4,269 | 3,518 | 3,570 | 4,207 | 4,499 | 4,651 | 4,807 | 5,461 |
| Average no. of prescription per patient with a prescription | 7.6 | 6.1 | 0.6 | 6.4 | 6.5 | 6.4 | 6.2 | 6.7 |
| Average no. of prescription per MG patient | 5.9 | 4.6 | 0.4 | 4.7 | 4.7 | 4.5 | 4.4 | 4.8 |
| **Indirect resource utilization** |  |  |  |  |  |  |  |  |
| **Welfare payment recipients** |  |  |  |  |  |  |  |  |
| No. of patients receiving disability pension for MG† | 116 | 120 | 111 | 114 | - | - | - | - |
| No. of patients receiving sick leave payments for MG (estimate)ǂ | 30 | 32 | 35 | 32 | 33 | 33 | 35 | - |
| No. of patients receiving work assessment allowance for MG (estimate) | 33 | 34 | 32 | 30 | 28 | 26 | 24 | 22 |

**†*Data not available for 2017-2020*.ǂ*Data not available for 2020. In 2020, the general population in Norway had an average of 2.8 GP visits per person, while the average number of emergency room visits was 0.2 ^28^.***

*Table S2: Health losses related to MG in Norway*

| **Non-fatal health losses** | |  |  |
| --- | --- | --- | --- |
| Quality of life MG patients | | 0.780 QALY | Mendoza et al (2020) ^17^ |
| Quality of life general population (same age) | | 0.811 QALY | Norwegian Medicines Agency (2020) ^16^ |
| Per patient QALY loss MG patients | | 0.031 QALY |  |
|  | |  |  |
|  | | **NPR** | **NorPD** |
| Number of patients (prevalence 12 Dec 2020) | | 1,141 | 1,072 |
| **Number of lost QALYs** | | **35.4** | **33.2** |
|  | |  |  |
| **Lost life years (premature death)** | |  |  |
| Average number of MG-related deaths per year | | 9.45 | Norwegian Cause of Death Registry (1996-2019) |
|  | |  |  |
| **Age group** | **Number of deaths per year (average 1996 – 2019) (NCDR)** | **Expected remaining QALYs by age group (Norwegian Medicines Agency (2020) ^16^** | **Estimated QALY loss** |
| 35-39 years | 0.04 | 41.3 | 1.72 |
| 40-44 years | 0.04 | 33.7 | 1.40 |
| 45-49 years | 0.04 | 29.7 | 1.24 |
| 50-54 years | 0.04 | 25.7 | 1.07 |
| 55-59 years | 0.13 | 22.0 | 2.75 |
| 60-64 years | 0.54 | 18.3 | 9.91 |
| 65-69 years | 0.42 | 14.9 | 6.21 |
| 70-74 years | 0.75 | 11.6 | 8.70 |
| 75-79 years | 1.33 | 8.7 | 11.60 |
| 80-84 years | 2.79 | 6.0 | 16.75 |
| 85-89 years | 2.17 | 4.1 | 8.88 |
| 90+ years | 1.17 | 2.8 | 3.27 |
| **Annual number of QALYs lost** | |  | **73.5** |

*Table S3: Number of patients† with prescription fills of the five most common pharmaceuticals to treat MG: from NorPD, 2013-2021*

| ***Drug***  ***Year*** | **Pyridostigmine**  **(N07AA02)** | **Prednisolone (H02AB06)** | **Azathioprine (L04AX01)** | **Mycophenolic acid**  **(L04AA06)** | **Ciclosporin**  **(L04AD01)** |
| --- | --- | --- | --- | --- | --- |
| **2013** | 561 | 320 | 167 | 22 | 9 |
| **2014** | 580 | 339 | 167 | 23 | 11 |
| **2015** | 591 | 346 | 175 | 23 | 9 |
| **2016** | 653 | 377 | 180 | 24 | 8 |
| **2017** | 688 | 409 | 204 | 25 | 8 |
| **2018** | 731 | 418 | 202 | 31 | 8 |
| **2019** | 774 | 433 | 208 | 33 | 8 |
| **2020** | 814 | 456 | 200 | 36 | 7 |
| **2021** | 837 | 470 | 210 | 40 | 5 |

^†^MG-patients who met the inclusion criteria for the NorPD study population, i.e., had at least three MG-specific drug prescriptions (pyridostigmine) or three MG-specific reimbursement codes (ICD-10 G70.0)

*Table S4: Number of patients diagnosed with MG in NPR alive by the end of each year† 2013-2021 according to different MG criteria*

| **Criteria**  **Year** | **At least one G70.0 as main diagnosis** | **At least one G70.0 diagnosis, main or contributory** | **At least two G70.0 diagnosis, main or contributory** | **At least two G70.0 diagnosis as main diagnosis** |
| --- | --- | --- | --- | --- |
| **2013** | **763** | **854** | **719** | **657** |
| **2014** | **825** | **914** | **760** | **693** |
| **2015** | **924** | **1,013** | **832** | **755** |
| **2016** | **1,007** | **1,106** | **900** | **816** |
| **2017** | **1,093** | **1,200** | **964** | **873** |
| **2018** | **1,189** | **1,297** | **1,029** | **935** |
| **2019** | **1,273** | **1,377** | **1,093** | **987** |
| **2020** | **1,343** | **1,446** | **1,141** | **1,032** |
| **2021** | **1,361** | **1,454** | **1,126** | **1,007** |

†Point prevalence by 31 Dec.

*Table S5: Number of patients diagnosed with MG in NorPD alive by the end of each year† 2013-2021 according to different MG criteria*

| **Criteria**  **Year** | **At least one or more pyridostigmine prescriptions or one or more G70.0 reimbursement codes** | **Two or more pyridostigmine prescriptions OR two or more G70.0 reimbursement codes** | **Three or more pyridostigmine prescriptions OR three or more G70.0 reimbursement codes** |
| --- | --- | --- | --- |
| **2013** | **816** | **725** | **687** |
| **2014** | **876** | **761** | **717** |
| **2015** | **955** | **824** | **772** |
| **2016** | **1,047** | **893** | **834** |
| **2017** | **1,148** | **972** | **900** |
| **2018** | **1,249** | **1,054** | **975** |
| **2019** | **1,341** | **1,125** | **1,036** |
| **2020** | **1,456** | **1,194** | **1,096** |
| **2021** | **1,539** | **1,243** | **1,134** |

†Point prevalence by 31 Dec.

*Table S6: List of MG-related drugs included in prescription drug cost analysis*

| **ATC-code** | **Drug name** |
| --- | --- |
| L01XC02 | Rituximab |
| L04AA25 | Eculizumab |
| N07AA01 | Neostigmine |
| N07AA02 | Pyridostigmine |
| N07AA30 | Ambenonium |
| N07AA03 | Distigmine |
| N07AA51 | Neostigmine, combinations |
| L04AX01 | Azathioprine |
| L04AA06 | Mycophenolic acid |
| L04AD01 | Ciclosporin |
| L04AX03 | Methotrexate |
| L04AD02 | Tacrolimus |
| L04AA13 | Leflunomide |
| L01AA01 | Cyclophosphamide |
| H02AB07 | Prednisone |
| H02AB06 | Prednisolone |

*Figure S1: Yearly cost per patient of MG-related hospital episodes, 2020*

| 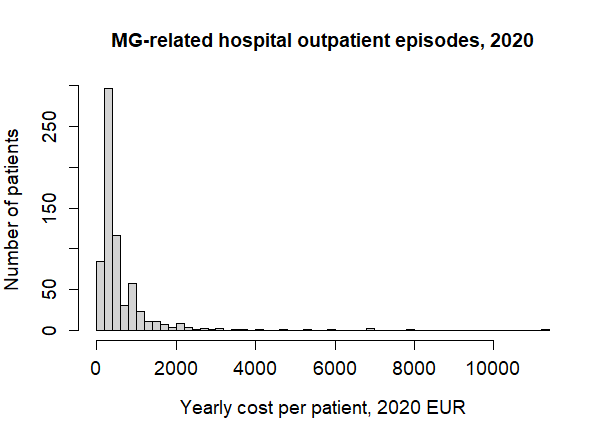 | 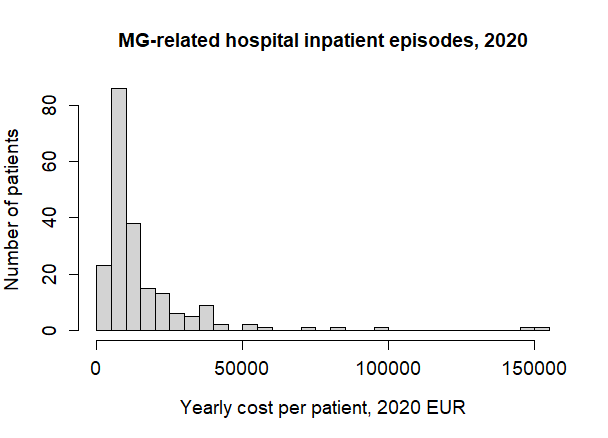 |
| --- | --- |
